# Mechanisms Mediating Anti-Inflammatory Effects of Delta-Tocotrienol and Tart Cherry Anthocyanins in 3T3-L1 Adipocytes

**DOI:** 10.3390/nu12113356

**Published:** 2020-10-30

**Authors:** Lexie Harlan, London T. Mena, Latha Ramalingam, Shasika Jayarathne, Chwan-Li Shen, Naima Moustaid-Moussa

**Affiliations:** 1 Obesity Research Institute and Department of Nutritional Sciences, Texas Tech University, Lubbock, TX 79409, USA; lexie.harlan@ttuhsc.edu (L.H.); london.t.mena@ttuhsc.edu (L.T.M.); latha.ramalingam@ttu.edu (L.R.); shasika-har.udahawatte@ttu.edu (S.J.); leslie.shen@ttuhsc.edu (C.-L.S.); 2 Center of Excellence for Integrative Health, Texas Tech University Health Sciences Center, Lubbock, TX 79430, USA; 3 Department of Pathology, School of Medicine, Texas Tech University Health Sciences Center, Lubbock, TX 79430, USA

**Keywords:** tart cherry, tocotrienol, obesity, 3t3-l1 adipocytes, inflammation

## Abstract

Chronic low-grade inflammation is a primary characteristic of obesity and can lead to other metabolic complications including insulin resistance and type 2 diabetes (T2D). Several anti-inflammatory dietary bioactives decrease inflammation that accompanies metabolic diseases. We are specifically interested in delta-tocotrienol, (DT3) an isomer of vitamin E, and tart cherry anthocyanins (TCA), both of which possess individual anti-inflammatory properties. We have previously demonstrated that DT3 and TCA, individually, reduced systemic and adipose tissue inflammation in rodent models of obesity. However, whether these compounds have combinatorial effects has not been determined yet. Hence, we hypothesize that a combined treatment of DT3 and TCA will have great effects in reducing inflammation in adipocytes, and that these effects are mediated via the nuclear factor kappa-light-chain-enhancer of activated B cells (NFkB), a major inflammatory transcription factor. We used 3T3-L1 adipocytes and treated them with 1–5 µM doses of DT3 along with tart cherry containing 18–36 µg anthocyanin/mL, to assess effects on inflammation. Neither DT3 nor TCA, nor their combinations had toxic effects on adipocytes. Furthermore, pro-inflammatory markers interleukin-6 (IL-6) and p-65 (subunit of NFkB) were reduced at the protein level in media collected from adipocytes with both individual and combined treatments. Additionally, other downstream targets of NFkB including macrophage inflammatory protein 2 (*Mip2*), and Cyclooxygenase-2 (*Cox2*) were also significantly downregulated (*p* ≤ 0.05) when treated with individual and combined doses of DT3 and TCA with no additional combinatorial effects. In summary, DT3 and TCA individually, are beneficial in reducing inflammation with no additional combinatorial effects.

## 1. Introduction

Prevalence of obesity is increasing at alarming rates with approximately 42% of adult Americans affected and obesity rates are comparable between men and women and between all ages [1,2]. Obesity leads to other metabolic complications including cardiovascular disorders and type 2 diabetes (T2D). One of the hallmarks of obesity is chronic low-grade inflammation that is triggered largely by adipocyte dysfunction [3,4,5]. The latter results in increased production and secretion of pro-inflammatory cytokines such as interleukin 6 (IL-6), tumor necrosis factor alpha (TNF-α), and monocyte chemoattractant protein 1 (MCP-1) [6,7] from adipose tissue. This in turn induces infiltration of macrophages, which further increases secretion of pro-inflammatory cytokines (IL-6, MCP-1, and TNF-α) from adipose tissue that aggravates obesity-associated inflammation and metabolic disorders.

Inflammatory cytokines produced during obesity activates receptors, which activate downstream NFkB pathway and other downstream inflammatory targets, such as macrophage inflammatory protein 2- alpha (*Mip2*) and cyclooxygenase-2 (*Cox2*) [8,9]. In addition to NFkB, JNK/ERK½ is another important inflammatory pathway that upregulates Activator Protein 1 (AP1) complex which aids in the transcription of nuclear activator of T cells-2 (NFAT2) and E26 transformation-specific transcription factor (ELK1) [10].

Various interventions are known to combat obesity, such as lifestyle (diet and exercise) behavioral interventions, as well as pharmacological treatments and bariatric surgery [11,12,13,14]. In recent years, dietary bioactive compounds and botanicals have been used to alleviate obesity and its complications. Among these, we focused on the effect of two bioactive compounds, namely a specific isoform of vitamin E, known as delta-tocotrienol (DT3), and anthocyanin-rich tart cherry.

Vitamin E, comprised of 4 isoforms each of tocopherols and tocotrienols (α,β,δ,γ), has been extensively studied for its anti-inflammatory effects [15,16,17,18,19]. Related to our current study, tocotrienols, T3′s, exhibit anti-inflammatory properties in both in vitro and in vivo studies [15,16,17,18,19]. In addition, our lab has demonstrated previously that DT3 reduced adiposity, inflammation in WAT and improved glucose clearance in diet induced obese mice [20]. However, the mechanisms behind its inflammation-reducing effects are not well understood.

Our second compound of interest is tart cherry anthocyanins (TCA), also known for their anti-inflammatory and antioxidant effects [21,22,23,24,25,26,27]. Anthocyanins belong to the flavonoid group and mostly found in fruits and vegetables [21,24]. Tart cherry has the greatest content of anthocyanins compared to other cherries and berries [28,29]. It has claimed health benefits for treatment of inflammatory diseases including arthritis and joint pain, as well as obesity [23,25,27]. Thus, both DT3 and TCA exert strong anti-inflammatory properties. However, whether their effects will be further enhanced when used in combination has not been previously studied. Accordingly, we hypothesized that DT3 and TCA will reduce adipocyte inflammation in part by inhibiting the NFkB pathway and/or the JNK/ERK½ pathway and that combined bioactives will have greater effects compared to individual compounds.

## 2. Materials and Methods

### 2.1. 3T3-L1 Cell Culture

3T3-L1 cells (ATCC, Washington, DC, USA) were incubated in Dulbecco’s Modified Eagle Medium (DMEM) (4.5 g/L D-Glucose, L-Glutamine, 110 mg/L Sodium Pyruvate; Gibco, Thermo Fisher Scientific, Waltham, MA, USA) with 10% fetal bovine serum (Atlanta Biologicals, Flowery Branch, GA, USA) and 1% antibiotics (50 U/mL penicillin, and 50 g/mL streptomycin; Thermo Fisher Scientific, Waltham, MA, USA). Upon confluence, cells were differentiated as described [30].

### 2.2. Bioactive Compound Preparation

Delta Gold, kindly provided by American River Nutrition, was utilized in our preparation of DT3. First, the density of the Delta Gold solution was calculated by weighing an empty 1.5 mL tube and then adding 1 mL of Delta Gold tube and reweighing. Then, this density of Delta Gold was used to calculate the density of the T3′s in it, approximately 90% tocotrienols. This density was then used to calculate the density of the DT3′s which is 88% of the 95% T3′s in the Delta Gold. With this density of the DT3 in Delta Gold and the molecular weight, we were able to make a solution of 5 mM concentration diluted in 100% ethanol to treat cells.

Frozen tart cherries were provided by Cherry Marketing Institute (Dewitt, MI). Tart cherries were crushed using a mortar and pistol, filtered using a 0.22 µm filter unit under the hood and centrifuged in a sterile tube to collect the supernatant, which is kept stored at −80 °C, as we have previously reported [31]. TC extract composition was measured using high performance liquid chromatography mass spectrometry (LC-MS) as we and others previously reported [31,32,33,34]. 100 g of tart cherries contain approximately 12.5–25.0 mg of anthocyanins, and 1 µL TC extract contains ~3 µg anthocyanins. Specifically, the TC extract from frozen TC contains 12,665 ± 1321 µg/g total phenolics, and 533 ± 47 µg/g total anthocyanin. For cell culture treatment, TCA extract (juice) was diluted in DMEM culture media, such that the final concentration in DMEM where cells were maintained was 18 µg/mL (when 6 µL of extract is used per ml culture media). Similarly, when we used 12 µL (extract)/mL culture media, that is ~36 µg of anthocyanins/mL media [34]. Once collected post-treatment, the media was stored at −80 °C for further analyses.

### 2.3. Dose Response Treatment

3T3-L1 adipocytes were pre-treated with 0.0001–100 µM DT3 or 18–36 µg/mL TCA for 4 h. Following which, they were treated with 200 ng/mL Lipopolysaccharide (LPS) (Sigma-Aldrich, St. Louis, MO, USA) and their corresponding dose of DT3 and/or TCA for 18 h if performing quantitative reverse transcription polymerase chain reaction (RT-qPCR) or for 1 h for western blot analyses. We used one hour of LPS incubation to mimic inflammation in adipocytes as we tested post-translational modifications of p65, which occurs very fast. Previous studies from our lab established this, as well as published literature indicates that phosphorylation occurs fast and is significantly reduced overtime. We also tried 18 h incubation for western blot analyses, but LPS did not induce the phosphorylation of P65 as expected. Following treatments, media was collected, and cells harvested and stored at −80 °C.

### 2.4. MTT (3-(4,5-Dimethylthiazol-2-Yl)-2,5-Diphenyltetrazolium Bromide) Assay

Cells were treated with DT3 and/or TCA for 22 h, with each corresponding dose. MTT solution was prepared with 5 mg of Thiazolyl Blue Tetrazolium Bromide (Sigma, St. Louis, MO) per one mL of media creating a 10% MTT solution for each well. After aspirating old media, MTT solution was added to the media (1:10). Cells were incubated for three hours and thirty minutes until violet color was developed in live cells. After this, dimethyl sulfoxide (Fisher Scientific, Hampton, NH) was added to stop the reaction. Finally, the plates were measured at 570 nm absorbance with a 690 nm background using Cytation 3 image reader (Winooski, VT, USA) to measure cell viability.

### 2.5. Gene Expression

RNA was isolated from 3T3-L1 adipocytes using the Quick RNA mini-prep (ZYMO Research, CA, USA). Total RNA was reverse transcribed into cDNA using iScriptTM Supermix for qPCR (Bio-Rad Laboratories Inc., Hercules, CA, USA). RT-qPCR was performed with cDNA for amplification of Interleukin-6 (IL-6), Interleukin-10 (IL-10), Monocyte chemoattractant protein-1 (MCP1), Tumor necrosis factor-alpha (TNFα), p65, Macrophage inflammatory protein-2 (mip2), Cyclooxygenase-2 (cox2), Toll-like receptor 2 (TLR2), and Toll-like receptor-4 (TLR4) (Sigma-Aldrich, St. Louis, MO, USA) with 18S and TATA-box binding protein (TBP) as the control. The primers used are listed below.

Il-6 F: 5′-CTGCAAGAGACTTCCATCCAGTT-3′ R: 5′-AGGGAAGGCCGTGGTTGT-3′
Mcp-1 F: 5′-ACTTCTATGCCTCCTGCTCAT-3′ R: 5′-CCTGCTTGTGATTCTCCTGTAG-3′
p65 F: 5′-GAAGCACAGATACCACCAAGAC-3′ R: 5′-TCAGCCTCATAGTAGCCATCC-3′
Tlr4 F: 5′-AGTAGCACTGACACCTTCCTT-3′ R: 5′-GCCTTAGCCTCTTCTCCTTCA -3′
Tbp F: 5′-GCCTTCCACCTTATGCTCAG-3′ R: 5′-GTTGTTGCTGCTGCTGTTG-3′
Tlr2 F: 5′- GAGATGTGTCCGCAATCATAGTT-3′ R: 5′-AGTCCGCACCTCCTTGAA-3′
Mip-2 F: 5′-CGAAGTCATAGCCACACTCAAG-3′ R: 5′-CCAGACAGGTGCCATCAGA-3′
Cox-2 F: 5′-CCATTAGCAGCCAGTTGTCA-3′ R: 5′-CGGAAGAGCATCGCAGAG-3′
Nfat-2 F: 5′-CTACAGCCGCAGGTGAGT-3′ R: 5′-AGCAGATGGAAGTGGAGAAGAG-3′
Elk-1 F: 5′-TAGGCTGGAGGAGGTGGAT-3′ R: 5′-ACAAGTAGTGGAGACGAGTATAGC-3′

### 2.6. Protein Isolation

Matured 3T3-L1 adipocytes were homogenized with modified radio-immunoprecipitation assay (RIPA) buffer containing protease and phosphatase inhibitors (Sigma-Aldrich, St. Louis, MO, USA) to prepare total protein lysates. Samples were centrifuged, supernatant extracted, and protein concentrations were determined using Bradford reagent (Bio-Rad Laboratories, Inc., Hercules, CA, USA). Following this, proteins were separated using SDS Page analyses and then transferred to Immobilon-FL^®^ Transfer Membranes (Millipore, Burlington, MA, USA) for identification of proteins. The membrane was incubated with primary antibodies (P-p65, total-p65, P-JNK, total-JNK, or tubulin (Cell Signaling Technology, Danvers, MA, USA) overnight at 4 °C. Following this, their corresponding secondary antibody (Alexa Fluor 680 conjugated anti-mouse and Alexa Fluor Plus 790 conjugated anti-rabbit; ThermoFisher Scientific, Waltham, MA, USA) were used for one hour and membrane scanned using Odyssey^®^ CLx Imaging System (LI-COR Biosciences, Lincoln, NE, USA). The protein quantification was done using densitometric analyses in the LI-COR software.

### 2.7. ELISA

IL-6 concentration (pg/mL) of 3T3-L1 adipocyte media was measured using an enzyme-linked immunosorbent assay ELISA (RayBiotech, Norcross, GA, USA). We used 100 µL of the mouse IL-6 protein standard or the supernatant media from treated (TCA or control) cultured cells, which was incubated for 2.5 h with assay diluent provided by the manufacturer in the assay kit, followed by the addition of biotin antibody for an hour. Then, 100 µL of streptavidin was added for 45 min followed by TMB one step substrate and finally the stop solution. The plate was read at 450 nm using a plate reader.

### 2.8. Statistical Analyses

Results are presented as mean ± SEM. One-way ANOVA test followed by Tukey’s post-hoc test (*p* < 0.05) was used to compare variables between groups using Graph pad prism software version 8 from Graph Pad. Data from q-PCR assays were analyzed using the CFX Manager software (version 3.1) provided by Bio-Rad Laboratories, Inc. using 2-ΔΔCT method [35,36].

## 3. Results

### 3.1. Dose Response Effects of Delta-Tocotrienol on Cell Viability in Adipocytes

**Figure 1:**
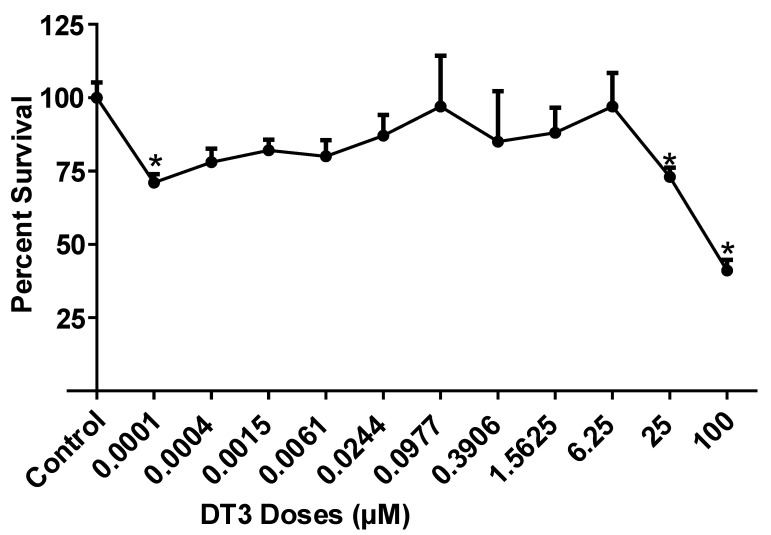
**Delta-tocotrienol does not reduce cell viability up to 25 µM.** Delta tocotrienol (DT3) were tested at doses ranging from 0.001–100 µM in 3T3-L1 adipocytes. DT3 at doses below 25 µM except 0.0001 µM had no difference in cell viability compared to control. *n* = 3, * *p* < 0.05.

DT3′s are generally recognized as safe (GRAS), hence we confirmed that DT3 is not toxic to adipocytes at most doses up to 25 µM. For this, we performed an MTT assay to determine DT3′s effects on cell viability. A lower dose of 0.0001 µM and high doses of 25 and 100 µM of DT3 significantly reduced cell viability compared to vehicle-treated control (*n* = 3 *p* < 0.05) (Figure 1), while other doses were not toxic to the cells.

**Figure 2:**
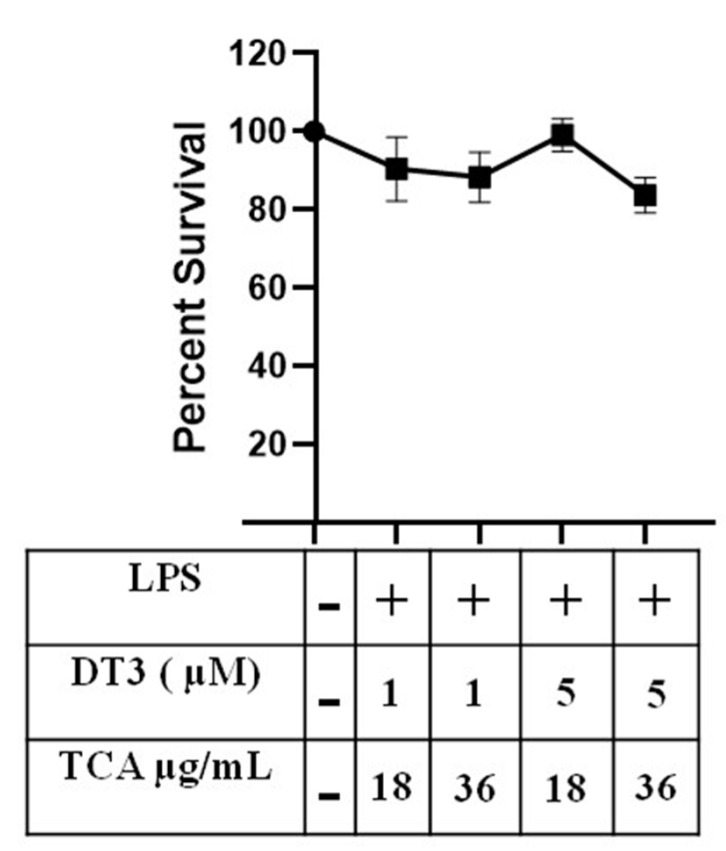
**Delta-tocotrienol and tart cherry anthocyanin (TCA) in combination are not toxic to cells.** DT3 1 µM + TCA 18 µg/mL DT3 1 µM + TCA 36 µg/mL, DT3 5 µM + TCA 18 µg/mL, DT3 5 µM + TCA 36 µg/mL DT3 and TCA were added to 3T3-L1 adipocytes to check viability. *n* = 2, *p* < 0.05.

Based on these MTT assays, we concluded that doses below 25 µM DT3 are safe to use for treating adipocytes. Additionally, our lab previously demonstrated that TCA doses up to 66 µL/mL did not negatively impact viability of 3T3-L1 cells [31]. Therefore, we next tested various doses of DT3 and TCA combinations on cell viability (DT3 1 µM + TCA 18 µg/mL, DT3 1 µM + TCA 36 µg/mL, DT3 5 µM + TCA 18 µg/mL, DT3 5 µM + TCA 36 µg/mL) to determine if combining them negatively impact our cells. As shown in Figure 2, we did not observe any toxic effects with any of the combination doses tested.

### 3.2. Delta-Tocotrienol Reduces IL-6 Secretion in Adipocytes

**Figure 3:**
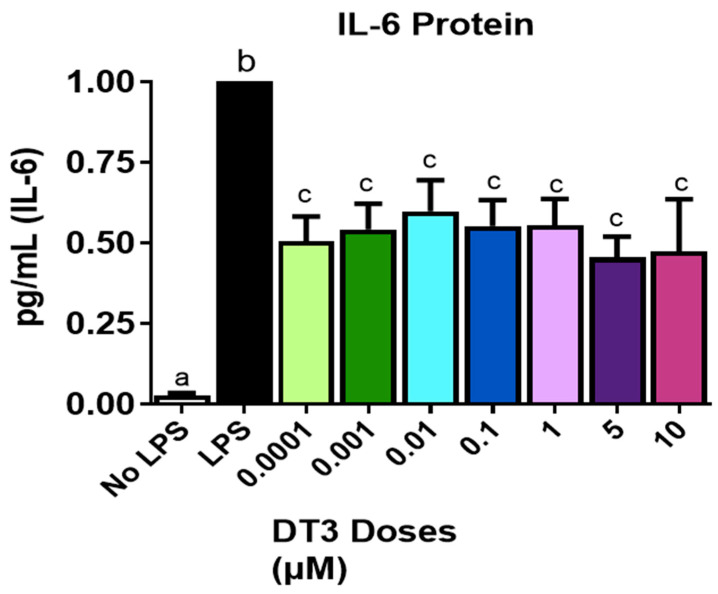
**Delta-Tocotrienol decreased IL-6 secretion:** All doses of DT3 significantly reduced IL-6 secretion measured in 3T3-L1 cells compared to the LPS control. Means with different letters (a, b, c) are significant. *n* = 6, *p* < 0.05.

Based on reductions on previously reported individual anti-inflammatory effects of DT3 and TCA, both in vivo and in vitro, we determined anti-inflammatory mechanisms of these compounds, individually, and in combination with LPS-stimulated adipocytes (to induce inflammation). As expected, LPS significantly increased IL-6 secretion from adipocytes after 24 h treatment. Moreover, all DT3 doses tested (up to 10 µM) significantly reduced IL-6 secretion from adipocytes, compared to LPS treatment, as shown in Figure 3 (*n* = 6, *p* < 0.05). Therefore, we chose 1 and 5 µM DT3 doses, consistent with the lowest doses used in previous studies for further experiments [20].

### 3.3. Delta-Tocotrienol and Tart Cherry Anthocyanins Reduce Expression of Genes Associated with the Inflammatory NFkB Pathway

**Figure 4:**
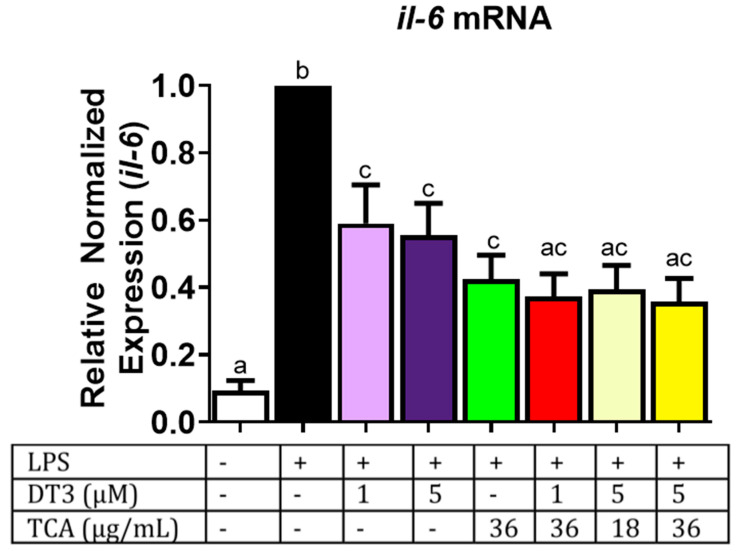
**Delta-tocotrienol and tart cherry anthocyanins decrease pro-inflammatory cytokine *IL-6*:** DT3 and TCA individually and in combination significantly reduced expression of *Il-6*. Means with different letters (a, b, c) are significant. *n* = 6, *p* < 0.05.

**Figure 5:**
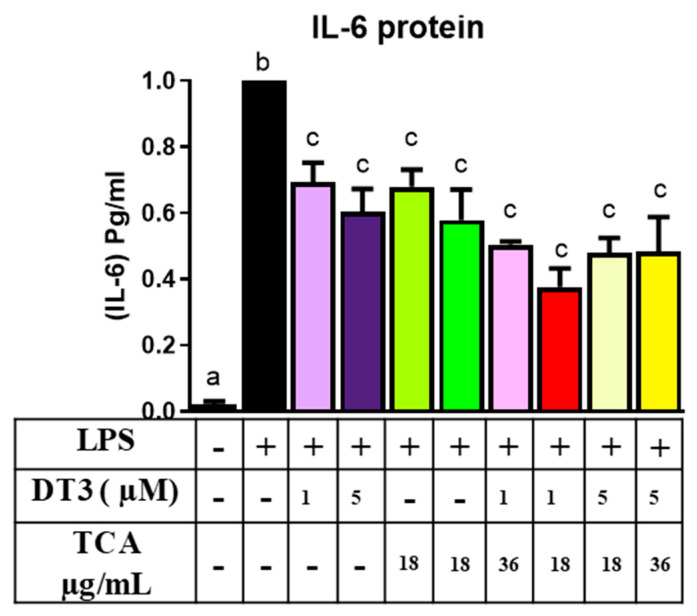
**Delta-tocotrienol and TCA decreased IL-6 secretion:** All doses of DT3 and TCA significantly reduced IL-6 secretion measured in 3T3-L1 adipocytes compared to LPS control Means with different letters (a, b, c) are significant. *n* = 5, *p* < 0.05.

In addition, based on our lab previously published data, we chose 18 and 36 µg/mL doses of TCA for further experiments [31]. Next, we wanted to determine if DT3 and TCA individually and in combination had anti-inflammatory potential by measuring changes in *Il-6* expression in adipocytes. DT3 and TCA, both individually and in combination, significantly reduced *Il-6* expression as shown in Figure 4 (*n* = 6, *p* < 0.05). The treatment groups all significantly reduced expression; however, no greater effect was seen with combinatorial doses than the individual doses. These results were also consistent with changes in secreted IL6 protein levels in media (Figure 5; *n* = 5, *p* < 0.05). Altogether, this data indicates that DT3 and TCA individually reduce IL-6 levels, but no additional combinatorial effects were observed. In addition, the IC50 for DT3 was ~1 µM which was in line with other studies where IC50 was ~2 µM [37].

**Figure 6:**
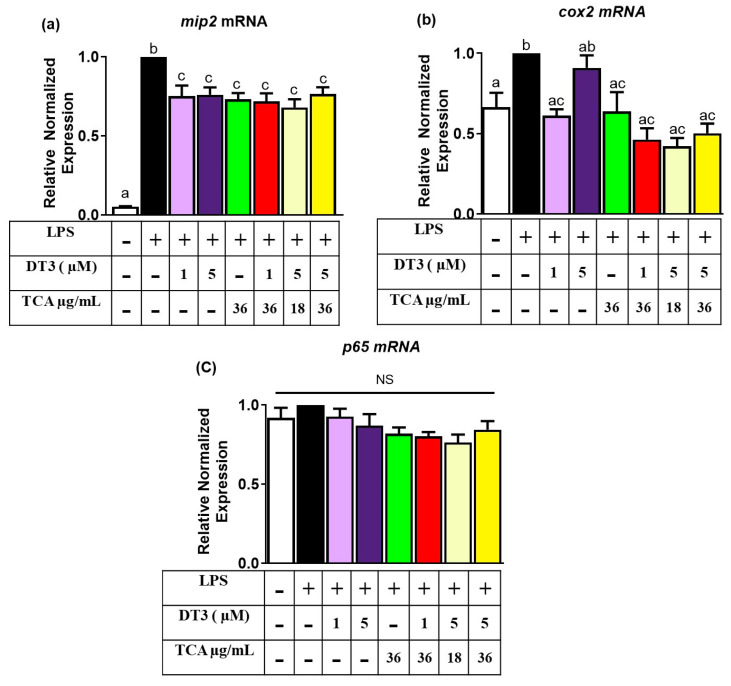
**Delta-tocotrienol and tart cherry anthocyanins decrease pro-inflammatory downstream transcripts of NFkB:** (**a**) DT3 and TCA individually and in combination significantly reduced expression of *Mip2* (**b**) *Cox2* (**c**) DT3 and TCA individually and in combination had no significant effects on expression of *p65*. Means with different letters (a, b, c) are significant. *n* = 6, *p* < 0.05.

We tested the downstream targets of the inflammatory NFkB (p65) and JNK pathways. These included *p65*, *Mip2*, *Cox2*, *Tlr-2*, *Tlr-4*, *Nfat2*, and *Elk1* genes. As expected, both *Mip2* and *Cox2* were significantly increased by LPS, compared to no LPS control (Figure 6a,b); *n* = 6, *p* < 0.05). *Mip2* expression was significantly decreased by all treatment groups compared to the LPS group (Figure 6a) (*n* = 6, *p* < 0.05). For *Cox2*, all treatments, excluding the 5 µM DT3 were significantly decreased compared to LPS group (Figure 6b) (*n* = 6, *p* < 0.05). There were no significant differences across any of the treatments or control, for the *p65* gene (Figure 6c; *n* = 6, *p* < 0.05). This was as expected, because p65 is post-transcriptionally regulated via phosphorylation and does not always change under mRNA level as shown in other studies.

**Figure 7:**
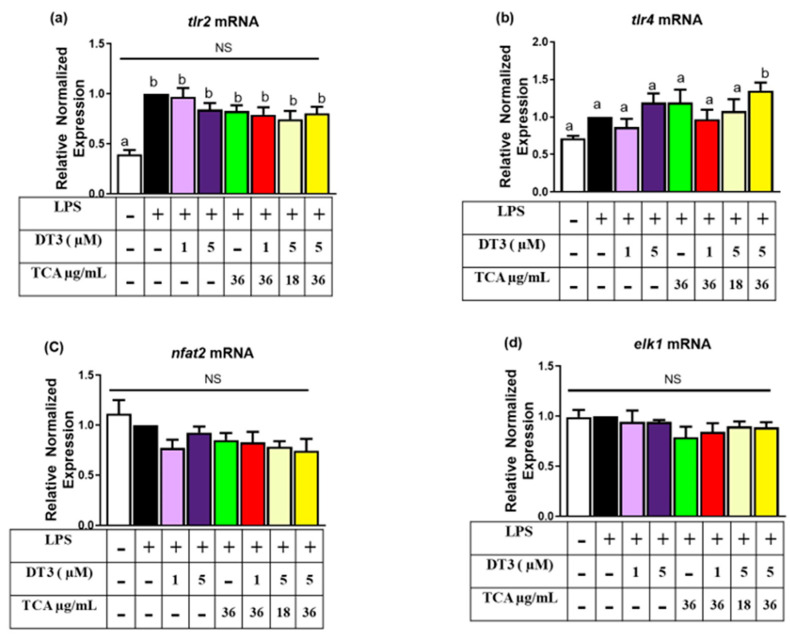
**Delta-tocotrienol and tart cherry anthocyanins showed no significant effect on *Tlr2*, *Tlr4*, *Nfat2*, and *Elk1*:** (**a**) DT3 and TCA individually and in combination showed no significant effect with any treatment group, compared to LPS for *Tlr2* gene. (**b**) DT3 and TCA combination at higher doses increased *Tlr4* levels. (**c**) No differences exist between any of the group for *Nfat2* and (**d**) *Elk1*. Means with different letters (a, b) are significant. *n* = 6, *p* < 0.05.

Finally, major inflammatory pathways such as those controlled by NFkB and JNK/ERK½ are downstream of receptor-mediated inflammatory pathways, we sought to determine changes in major inflammatory receptors, such as the toll-like receptor 2 and 4 (*Tlr2* and -4) gene expression. LPS significantly induced *Tlr2* but not *Tlr4* expression. There were no significant changes in *Tlr-2* (Figure 7a) or *Tlr4* (Figure 7b) gene expression with individual or combined treatments tested; however, the highest doses tested of DT3 and TCA unexpectedly upregulated *TLR4*, compared to LPS. (Figure 7b; *n* = 6, *p* < 0.05). We also tested nfat2 and elk1 but saw no significant difference in any of the treatment groups compared to the LPS and no LPS controls.

### 3.4. Delta-Tocotrienol and Tart Cherry Anthocyanins Downregulate NFkB and JNK/ERK½ Activation

**Figure 8:**
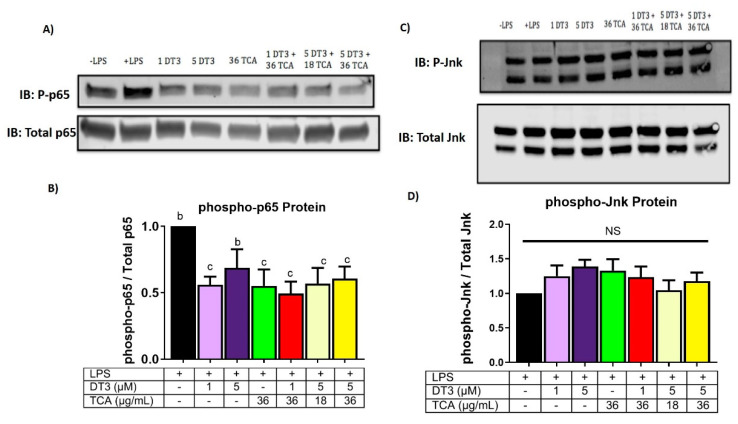
**Delta-tocotrienol and tart cherry anthocyanin reduced protein expression of p65 in NFkB pathway.** (**A**,**B**) DT3 and TCA significantly reduced p65 expression in all but the 5 µM dT3 treatment group, compared to the LPS control means with different letters (b, c) are significant. *n* = 10, *p* < 0.05. (**C**,**D**). DT3 and TCA showed no reduction in expression of JNK (*n* = 10).

As major inflammatory signaling pathways including NFkB and JNK/ERK are regulated post-transcriptionally, we determined next if DT3 and TCA reduced inflammation by inhibiting the NFkB or JNK pathways. Accordingly, we measure phosphorylated levels of key proteins in these pathways, namely p65 and JNK. All doses tested of DT3 and TCA reduced levels of P-p65 compared to the LPS control. Further, when considering phospho-P65/Total P65, all doses, except for the 1 µM DT3 dose, significantly reduced P65 protein levels (Figure 8a,b) (*n* = 10, *p* < 0.05). Lastly, none of the doses of DT3 and TCA reduced JNK/ERK½ pathway compared to the LPS control (Figure 8c,d), *n* = 10).

## 4. Discussion

Individually, both tocotrienols and anthocyanins [15,16,19,21] are known for their anti-inflammatory effects but their combinatorial effects have not been examined. Our lab previously demonstrated DT3 and TCA to exhibit anti-inflammatory effects. Due to their similar properties, we tested whether the combination of DT3 and TCA would enhance individual effects on adipocyte inflammation. We confirmed using adipocytes that DT3 and TCA each significantly reduced inflammation and did so at least in part through the NFkB pathway. However, we did not observe any combinational effects of the two compounds.

Previous studies have shown that T3s doses ranging from 0.024–25 µM dose-dependently reduced triglyceride accumulation and secretion of pro-inflammatory cytokines, and inhibited the NFkB pathways [38,39,40,41]. However, these studies use tocotrienol-rich fraction (TRFs), which contain all isoforms of T3s and alpha-tocopherol (αTOH), making it difficult to determine which isoform was most effective in reducing inflammation and the mechanism behind that reduction [38,39,40]. Tocotrienols share structural homology with tocopherols, but because of the unsaturation in the hydrophobic chain, they incorporate better into the membranes and demonstrate better functional properties [42]. Furthermore, we had determined previously that DT3 reduces inflammation in an in vivo model [16]. Based on this, we used DT3 isomer alone and found that it had comparable effects to TRF on inflammation and this was consistent with our previously reported anti-inflammatory effects of DT3 in diet-induced obese mice [20].

Gamma-tocotrienol, another isoform of vitamin E, has been studied for its anti-inflammatory properties. Matsunaga et al. showed that 2.4 µM γ-T3 reduced MCP-1 and IL-6 secretions in 3T3-L1 cells, while adiponectin secretion was restored [40], and further demonstrated that γT3 also reduced expression of IKB and NFkB [40]. Uto-kondo et al. showed that γT3 reduced triglyceride content in differentiating 3T3-L1 adipocytes, and inhibited adipogenesis to a greater effect than α-T3 [38]. This is in line with our study where we demonstrated DT3 to reduce inflammation suggesting some isomers have beneficial effects. Further, an interesting study tested different isomers of T3s and identified that only DT3 improved glucose tolerance and insulin sensitivity compared to other isomers and this was in part through inflammation which is consistent with our study [42]. Additionally, a study suggested that lipid synthesis was lower in adipocytes when treated with γT3 and DT3 individually [39]. Hence, in the future, it is worthy to test γT3 and DT3 combinatorial effects.

The second bioactive compound we tested was TCA, which also showed similar anti-inflammatory effects to those described above for DT3. Studies have shown that anthocyanins at doses of 2.5–50 µg/mL dose-dependently reduced adipocyte differentiation, lipid accumulation, lipolysis, and secretion of pro-inflammatory cytokines [43,44,45,46]. However, these studies used anthocyanins derived from sources other than tart cherries and thereby, make these results difficult to interpret for our study since C1G and C2G (the primary cyanidin in tart cherries) were not the prominent cyanidins in these sources [43,44,45,46]. A previous study in our lab using Zucker fatty rats showed decreased inflammatory markers such as *Il-6*, *Cd-11b*, *Mcp-1*, *iNOS*, and *Tnfα* in white adipose tissue [31]. Moreover, the same study confirmed TCA reduced inflammatory markers in adipocytes.

Bioactive compound research has become more prominent, with promising effects being discovered. This is why we chose to combine two already beneficial compounds in order to determine if they could have even greater effects (additive or synergistic) together. Hence, for this study, we investigated the combinatorial anti-inflammatory effects of TCA and DT3 and tested whether similar mechanisms mediate their effects.

In line with the previous study, we also found that TCA reduced NFkB individually and when combined with DT3, indicating that the mechanisms mediating effects of both compounds could be through the same NFkB inflammatory pathway. Furthermore, downstream targets of NFkB which include mip2 and cox2 were reduced by both TCA and DT3, but no combinational effects were observed. This could indicate that both these compounds use similar mechanisms to reduce inflammation. Further, we did not see any additional combinatorial effects due to probable competition between the two compounds. Another probability is that we are using a higher concentration of both the compounds and beneficial effects could be maxed out and lower doses need to be tested in the future. This is especially true for vitamin E analogues which tend to act as pro-oxidant at higher concentrations than as anti-oxidant [47]. Lastly, TCA is a water-soluble bioactive compound, while DT3 is a fat-soluble bioactive compound, hence synergistic activity was not found. This is similar to studies which combine green tea polyphenols (water-soluble) and DT3 (fat-soluble) [48].

Several other studies determined the combined effects of bioactive compounds. One such study combined myrrh and frankincense (both derived from plants) to reduce hind paw inflammation/edema in rats, which was effective at doses ranging from 10 to 76.92 mg/kg [49]. Another study by Rinwa et al. combined black-pepper derived piperine (20 mg/kg) and Curcumin (100, 200 mg/kg), and reported reduced TNF-a brain levels and caspase-3 levels by these compounds [50].

Clinical studies are a necessary step moving forward, so as to truly determine the physiological effects of tocotrienol and TCA on humans. One such study [51], showed that tocotrienol has generally been marked as safe across human dosages. Further, a recent study showed that DT3 up to 600 mg/day was safe for kidney and liver function [52]. The major concern was bleeding, as tocotrienols have anti-coagulant properties, but as mentioned before almost all the trials showed safe dosages. In two studies [51,53], tocotrienols were detectible in the blood plasma after supplementation, showing that the body is absorbing this compound and it was detectible in the vital organs. It is also noted that anthocyanins (both from sweet and tart cherry), reduce oxidative stress and improve metabolic markers in human clinical studies, as is reflective of our study in cell culture [54,55]. Additionally, anthocyanins show anti-inflammatory effects, promotion of healthy glucose regulation, anti-arthritic effects, and decreased liver triglycerides [54]. Most of the clinical studies used tart cherry juice which is comparable to the tart cherry extract we used in our cell culture studies.

## 5. Conclusions

In conclusion, DT3 and TCA each exert potent anti-inflammatory effects, which are mediated through the NFKB pathway. Moreover, combining DT3 and TCA does not enhance their individual effects, and reduction in inflammatory targets including NFkB was comparable for the individual and combined doses, suggesting that these compounds may act through similar mechanisms of action (at least in reducing inflammation through NFkB). We only focused on the downstream targets of JNK (nfat2 and elk1) in gene expression studies, so we do not have a full understanding of this whole pathway. More studies are needed to fully determine mechanisms of DT3 and TCA through the JNK and MAPK pathways. As both DT3 and TCA’s are available dietary compounds, they provide excellent safe options for reducing obesity-associated inflammation. Additional animal and clinical studies are warranted to further understand mechanisms mediating the protective effects of these compounds in metabolic diseases.
